# MXene: A wonderful nanomaterial in antibacterial

**DOI:** 10.3389/fbioe.2024.1338539

**Published:** 2024-02-01

**Authors:** Surong Ye, Huichao Zhang, Huiyan Lai, Jingyu Xu, Ling Yu, Zitong Ye, Luyi Yang

**Affiliations:** ^1^ Department of Orthodontics, Hospital of Stomatology, Jilin University, Changchun, China; ^2^ Stomatology College of Chifeng University, Chifeng, China; ^3^ College of Chemistry and Chemical Engineering, Xiamen University, and Discipline of Intelligent Instrument and Equipment, Xiamen, China

**Keywords:** MXene, antibacterial, nanomaterial, bactericidal, photothermal therapy

## Abstract

Increasing bacterial infections and growing resistance to available drugs pose a serious threat to human health and the environment. Although antibiotics are crucial in fighting bacterial infections, their excessive use not only weakens our immune system but also contributes to bacterial resistance. These negative effects have caused doctors to be troubled by the clinical application of antibiotics. Facing this challenge, it is urgent to explore a new antibacterial strategy. MXene has been extensively reported in tumor therapy and biosensors due to its wonderful performance. Due to its large specific surface area, remarkable chemical stability, hydrophilicity, wide interlayer spacing, and excellent adsorption and reduction ability, it has shown wonderful potential for biopharmaceutical applications. However, there are few antimicrobial evaluations on MXene. The current antimicrobial mechanisms of MXene mainly include physical damage, induced oxidative stress, and photothermal and photodynamic therapy. In this paper, we reviewed MXene-based antimicrobial composites and discussed the application of MXene in bacterial infections to guide further research in the antimicrobial field.

## 1 Introduction

One of the most critical challenges in wound healing is bacterial infection, affecting millions of people worldwide. Antibiotics are the main strategy currently used for the therapy of bacterial infections. The overuse of antibiotics not only damages the immune system but also results in bacterial resistance (such as *Staphylococcus aureus* (*S. aureus*), *Escherichia coli* (*E. coli*), *Klebsiella pneumoniae* (*K. pneumoniae*), *Acinetobacter* baumannii (*A. baumannii*), *Pseudomonas aeruginosa* (*P. aeruginosa*), and *Streptococcus* pneumoniae (*S. pneumoniae*)) (Turner et al., 2019; Gao et al., 2021; Pino et al., 2023). Multidrug-resistant bacteria are a global crisis, increasing incidence rate and mortality in patients with infections (Wang et al., 2018; Xie et al., 2023). According to estimates by the World Health Organization, antibiotic-resistant bacteria are responsible for nearly 7 million deaths per year globally, and this number is expected to skyrocket to 10 million in 2050 if effective treatments are not found and if this trend continues (Li et al., 2023). Infections caused by drug-resistant bacteria prolong hospitalization, and the annual societal cost of treating antibiotic-resistant infections is estimated to be $20 billion, posing a significant economic burden on global healthcare systems (Naylor et al., 2018). What’s more, the large-scale use of antibiotics increases the risk of environmental contamination (Zhu et al., 2017). Multidrug-resistant or extensively drug-resistant pathogens are causing a growing number of bacterial infections in clinical practice around the world (Christaki et al., 2020). In addition to antibiotic resistance, recalcitrant biofilm formation is another vexing problem. Biofilm formation on the surface of medical devices or implants exhibits strong resistance to conventional antibiotics, resulting in persistent and chronic polymer-related infections, which have always been a serious practical threat (Costerton et al., 1999; Verboni et al., 2023). Therefore, an urgent need is to explore and design a new strategy to combat drug-resistant bacterial infections.

Rapid advances in materials science and nanotechnology have enabled the development of new materials and methods for treating bacterial infections, making strategies to overcome bacterial resistance increasingly important (Zhu et al., 2023). Nanomaterials can interact with microorganisms without relying on antibiotics through multivalent interaction (multivalent interaction) to inhibit cellular functions (Fu et al., 2019; Pashazadeh-Panahi and Hasanzadeh, 2019; Yu et al., 2020). Two-dimensional (2D) materials such as graphene, black phosphorus (BP), transition metal sulfide (TMDC), hexagonal boron nitride (hBN), and graphitic carbon nitride have been successfully used as novel antimicrobial agents or biological applications by their excellent electrical conductivity, mechanical properties, and remarkable properties in fighting bacterial infections (Novoselov et al., 2004; Wang et al., 2020a; Tan et al., 2020; Yang et al., 2023). However, these materials also have certain party limitations, such as graphene lack of bandgap and visible region light absorption (Wang et al., 2012). MoS_2_, although correcting graphene’s zero bandgap weakness, has a relatively low charge carrier mobility. TMDC has a large bandgap and strong light absorption but relatively low charge carrier mobility. Black phosphorus has a high carrier mobility but poor material stability, and strong dipole-dipole interactions between water molecules and phosphorus lead to significant distortions in the black phosphorus structure (Lee et al., 2016). Hence, scholars are searching for a more perfect 2D nanomaterial that can decrease or eradicate the constraints mentioned above in the field of biomedical applications.

Transition metal carbides, nitrides, and carbonitrides, termed MXene, have excellent photothermal and catalytic properties under near-infrared irradiation, making them a promising candidate for drug-free antibacterial treatment (Yu et al., 2022). MXene is commonly prepared by chemically etching a bulk phase known as a MAX phase, followed by mechanical stripping and other processes (Naguib et al., 2014; Lim et al., 2021). With its modifiable chemical structure and unique characteristics, MXene has attracted great interest in the research of 2D nanomaterials. Since the first ever MXene was exfoliated from 3D titanium aluminum carbide (Ti_3_AlC_2_), producing 2D titanium-carbide (Ti_3_C_2_) layers (Naguib et al., 2011), experimental and theoretical studies on MXene materials have mushroomed, rapidly expanding into an extensive system of 2D materials (Gogotsi and Anasori, 2019). So far, researchers have successfully synthesized more than 30 MXene materials with different compositions, and theoretical calculations predict the existence of more than 70 MXene materials (Huang et al., 2020). Compared with other 2D nanomaterials, due to the transition metal carbides and abundant functional groups (e.g., hydroxyl, oxygen), MXene combines the advantages of traditional 2D nanomaterials with some unique properties (Turner et al., 2019): MXene has excellent electronic conductivity. They exhibit strong absorption and maintain high photothermal conversion efficiency in both the first and second near-infrared biological windows, which is promising for applications in photothermal therapy. And they also exhibit strong absorption in the ultraviolet range due to interband jumps (Liu et al., 2019; Lin et al., 2021), whereas the absorbance of conventional two-dimensional nanomaterials, MoS_2_ (Cheng et al., 2014), black phosphorus (Sun et al., 2015), and graphene (Yang et al., 2010; Robinson et al., 2011), decreases significantly in the second NIR biological window (Pino et al., 2023). MXene has excellent hydrophilicity. A large number of hydrophilic surface functional groups exist on the surface of MXene, which is conducive to stable interactions with water molecules, in contrast to graphene, which has strong hydrophobicity, is poorly dispersed in water, and requires complex surface modification before it can be helpful to in aqueous environments (Chou et al., 2013; Gao et al., 2021) MXene has excellent mechanical properties, and its hardness (Ti_2_CT_x_: 5.2 eV; Ti_3_C_2_T_x_: 49.5 eV; Ti_4_C_3_Tx: 47.4 eV) (Borysiuk et al., 2018) exceeds that of graphene (2.3 eV) (Kang and Lee, 2013) and MoS_2_ (9.61 eV) (Jiang et al., 2013). The Young’s modulus of monolayer Ti_3_C_2_T_x_ is 0.33 ± 0.03 TPa (Lipatov et al., 2018), which is one-third of that of graphene (1.02 ± 0.03 TPa) (Lee et al., 2008) and higher than that of GO (0.208 ± 0.023 GPa) (Suk et al., 2010) and MoS_2_ (0.27 ± 0.1 TPa) (Bertolazzi et al., 2011). In addition, some of the shortcomings of MXene *in vivo*, including poor aqueous dispersion and slow degradation rate, can be effectively improved by surface modification and functionalization without compromising its properties, broadening its application in biomedical fields (Lin et al., 2018). In recent years, MXene has become a focus point in antibacterial drug research.

**FIGURE 1:**
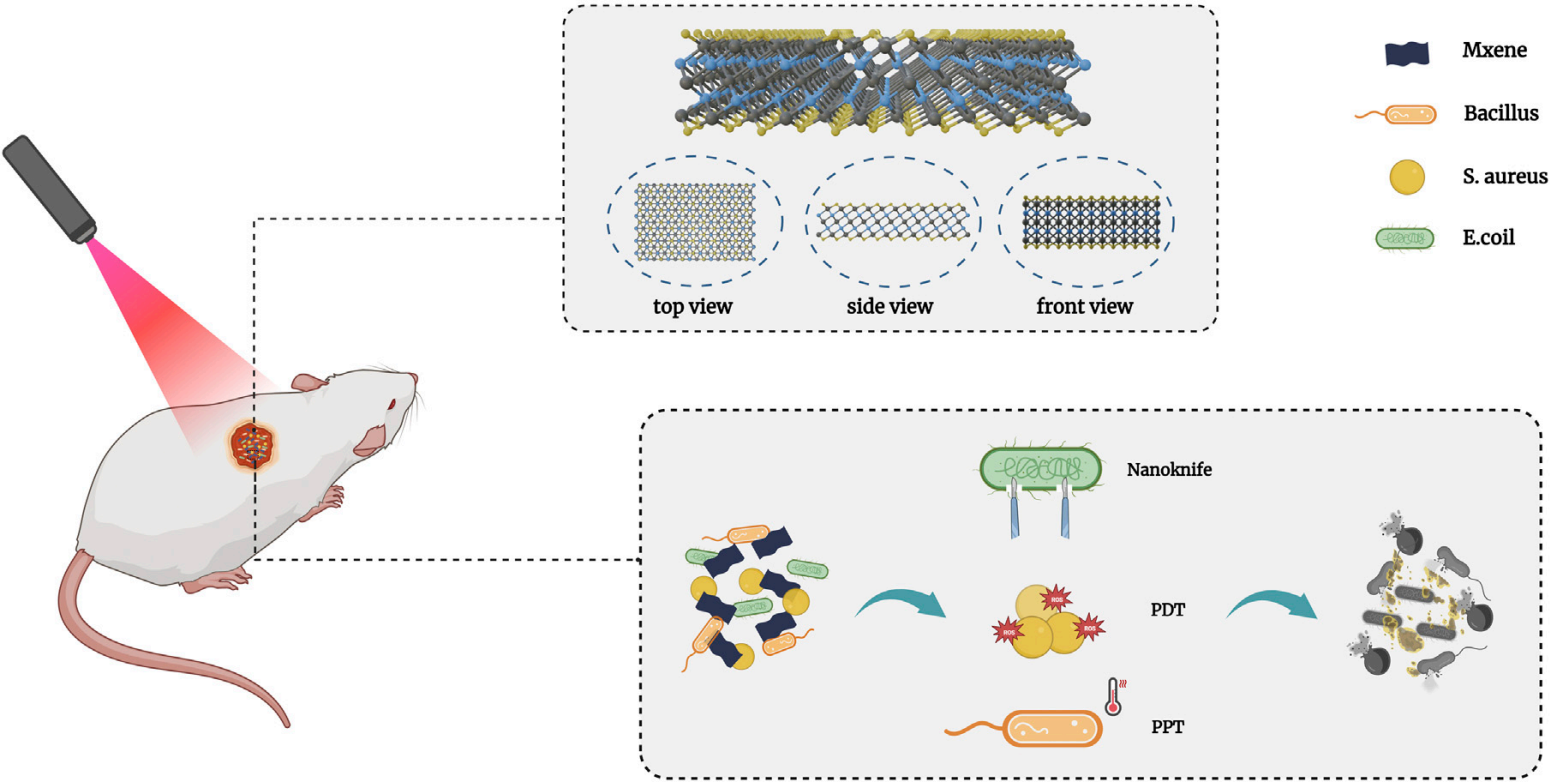
Characteristic and antibacterial mechanism of MXene.

To date, the biomedical applications of MXene, especially in biosensing, antitumor, and disease diagnostic imaging, have been well reviewed (Iravani and Varma, 2022; Amara et al., 2023; Gao et al., 2023). However, there are limited relevant reviews on the research progress in the field of MXene antimicrobials. This paper presents an overview of the recent advances of MXene in the antimicrobial field, including the structure and synthesis, biosafety, antimicrobial mechanism, and various MXene-based composites in antimicrobial applications, intending to lay a theoretical foundation for future applications in the antibacterial field (Figure 1).

## 2 Structure and synthesis of MXene

### 2.1 Structure

**FIGURE 2:**
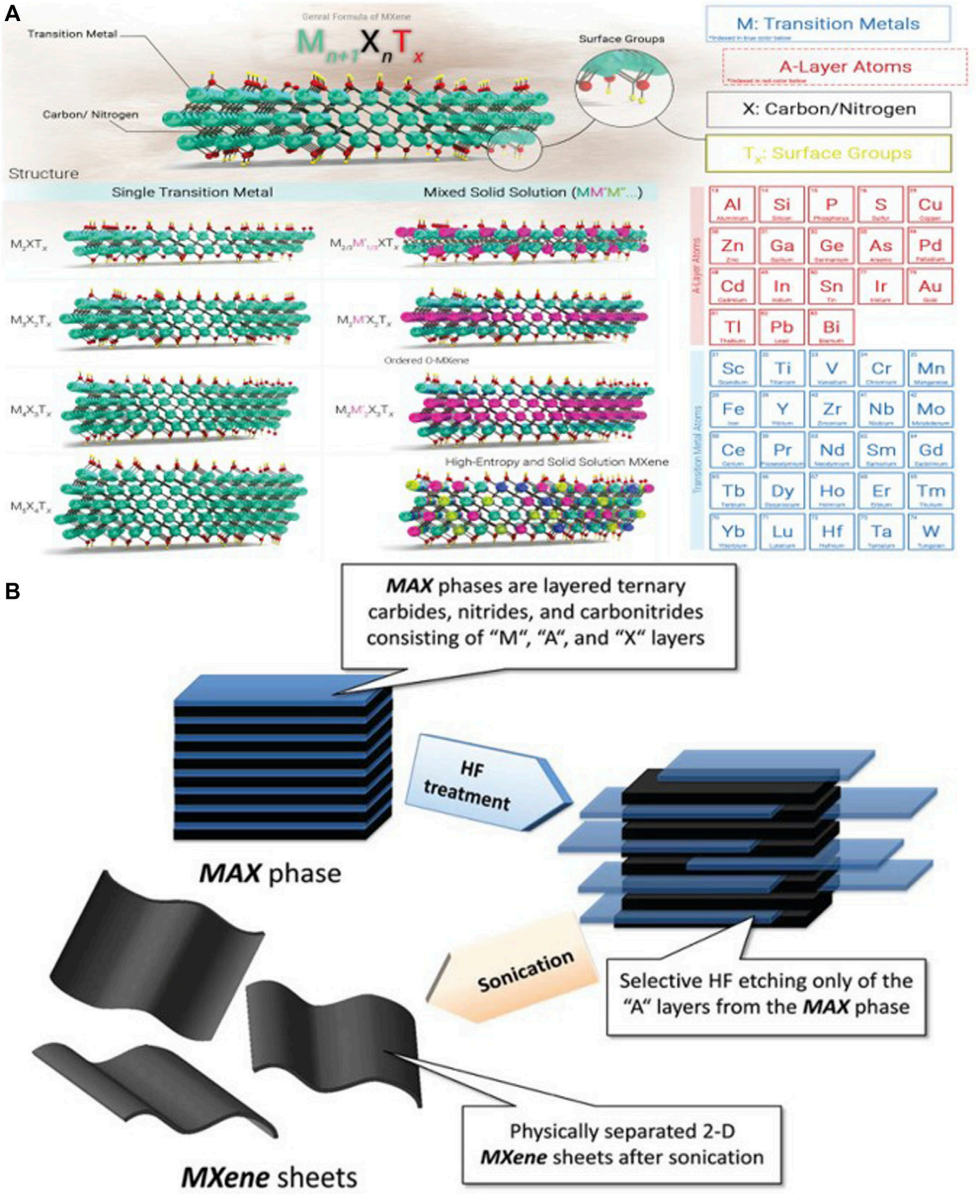
**(A)** Different structures of MXene and the possible elements that have been utilized for the fabrication of different MAX phases (Seidi et al., 2023). Adapted with permission from Babak Anasori, Huining Xiao, Chengcheng Li, et al. MXene Antibacterial Properties and Applications: A Review and Perspective. Copyright 2023, Wiley-VCH GmbH. **(B)** Schematic for the exfoliation process of MAX phases and formation of MXene (Naguib et al., 2012). Adapted with permission from Michael Naguib, Olha Mashtalir, Joshua Carle, et al. Two-Dimensional Transition Metal Carbides. Copyright 2012, American Chemical Society (ACS). American Chemical Society (ACS).

The ternary metal carbide or nitride MAX phase (M_n+1_AX_n_) is the precursor for the synthesis of MXene. The MAX phase is a layered hexagonal structure consisting of alternating stacks of M-layers and A-group elements, with X-atoms filling the octahedral voids of the M-layers (Li et al., 2022a). M represents early transition metal sites (e.g., Ti, Zr, V, Nb, and Mo), A represents elements of IIIA or IV A (e.g., Al, Si, Ga, and Ge), and X represents C or N. The chemical bonding between M and X exhibits strong covalent/metallic/ionic properties, whereas the chemical bonding between M and A has only metallic bonding properties. By taking advantage of this bond-energy phenomenon, the A layer can be selectively removed from the MAX phase by strong acids or molten salts using this bonding energy phenomenon, without destroying the M-X bonds (Naguib et al., 2014). After selective etching of the A layer, the A element is replaced by surface functional groups (Tx) (e.g., OH, O, F, and Cl) to form MXene (M_n+1_X_n_T_x_), and the presence of the surface functional groups not only improves the electrical conductivity but also increases the activity of the material, which provides a broad design space for further surface modification (Figure 2A) (Kuang et al., 2020; VahidMohammadi et al., 2021; Noor et al., 2023).

### 2.2 Synthesis

In the past few years, MXene synthesis methods have evolved rapidly to explore more MXene. In general, they can be divided into two categories: top-down methods, which are based on direct exfoliation of bulk crystals, and bottom-up methods, in which MXene preparation is controlled at the atomic/molecular level (Lin et al., 2021). Both methods successfully prepare monolayer, few-layer, and multilayer nanostructures of MXene.

The top-down method, which breaks down the crystal into monolayer MXene sheets, is the classical method for preparing MXene (Figure 2B) and consists of two stages: chemical etching and layering. Taking advantage of the difference in bonding energy between the M-A and M-X bonds, an etchant is used to selectively break the M-A bonds in the MAX phase and strip the A-layer atoms from MAX (Xing et al., 2018). The first reported method is HF etching, which can effectively remove the A layer in the MAX phase to generate OH and F surface terminated groups (Naguib et al., 2011). After etching, the layered bulk structure of the MAX phase is gradually transformed into a fluffy “accordion” structure, and 2D MXene nanosheets are obtained by layering MXene through mechanical ultrasonication or the weakening of the interlayer van der Waals force by the intercalating agent. The intercalating agents are generally organic base molecules (tetramethylammonium hydroxide (Xuan et al., 2016), tetrabutylammonium hydroxide (Halim et al., 2016)), polar organic molecules (dimethylformamide, dimethylsulfoxide (Mashtalir et al., 2013; Hussein et al., 2019)) or metal cations (Lukatskaya et al., 2013). The advantages of this method are lower reaction temperature, shorter etching time, simplicity and efficiency, which can be applied to various MXene compounds. However, HF is highly corrosive and toxic, which may cause excessive etching and reduce the performance of MXene. What’s more, HF residues induce cell death, which is detrimental to the application and development of MXene in biomedical fields (Alhabeb et al., 2017; Caffrey, 2018). In order to reduce the risk during the preparation process and improve the biosafety, researchers started to seek and develop safer and more effective etching systems, such as NH_4_HF_2_ (Halim et al., 2014), NH_4_F (Liu et al., 2017a), LiF/HCL (Ghidiu et al., 2014), and mild molten salt etching (Cao et al., 2018). Compared to HF, the *in situ* formation of hydrofluoric acid by mixing fluoride salts (e.g., LiF, KF, NaF, CsF, etc.) and hydrochloric acid provides milder, safer, and more efficient etching conditions, avoids the direct use of highly corrosive HF, and produces MXene crystals with fewer defects and larger layer gaps and transverse dimensions (Ghidiu et al., 2014; Alhabeb et al., 2017). More importantly, due to the presence of metal cations (e.g., Li^+^), single-layer or few-layer MXene can be obtained by hand-shaking method or mild ultrasonication without intercalation agent after etching, which greatly simplifies the preparation process and has gradually become a mainstream method for the preparation of MXene (Alhabeb et al., 2017). Recently, the use of algal extracts for intercalation and delamination (intercalate and delaminate) of MAX has been proposed to avoid the harsh conditions of traditional delamination methods. This method is green and high-yielding, but it should be noted that the by-products after etching will coexist with MXene, which may affect the performance of nanosheets (Zada et al., 2020).

In addition to the widely used top-down synthesis method, bottom-up synthesis has also been investigated. Unlike the strategy of preparing MXene materials by exfoliation from the layered MAX phase, the bottom-up method is a controlled synthesis of MXene at the atomic level. Layered structures are synthesized by crystal growth using individual inorganic atoms or molecules as precursors (Zhengyang et al., 2015). Bottom-up methods allow precise control of MXene parameters (e.g., composition, size, morphology, or surface groups) to prepare ultrathin sheets with large lateral dimensions, long interlayer distances, very low defect concentrations, and large specific surface areas (Verger et al., 2019; Rozmysłowska-Wojciechowska et al., 2020). Commonly used methods include Plasma-enhanced pulsed-laser deposition (PE-PLD) (Zhang et al., 2017), chemical vapour deposition (CVD) (Gogotsi, 2015; Xu et al., 2015) and Salt-Templated (Jia et al., 2017; Xiao et al., 2017). Due to the atomic and structural complexity of MXene, the internal structure and mechanism of intergroup interactions are still unclear (Zhong et al., 2022). Moreover, the preparation conditions of the bottom-up method are relatively harsh, usually requiring higher temperatures and longer etching times, making it challenging to produce on a large scale (Lin et al., 2021; Sana et al., 2023). There are limited studies on the biocompatibility of bottom-up synthesized MXene materials, which also restricts its promotion in the biomedical field (Soleymaniha et al., 2019). Overall, the preparation of MXene by bottom-up techniques remains challenging.

## 3 Biocompatibility and biodegradability

### 3.1 Biocompatibility

MXene-based materials are known to have many excellent properties, including large specific surface area, abundant surface functional groups, and excellent electronic, mechanical, physical and chemical properties (Li et al., 2015). However, in the biomedical field, biocompatibility, biodegradation, and toxicity of biomaterials are the most critical parameters to realize their potential. To meet clinical needs, long-term low toxicity, controlled biodegradability, and high biocompatibility are necessary.

In *vitro* toxicity assays, the toxic cytotoxicity of MXene is strongly related to its size, oxidation state, surface functional groups, synthesis method, administration route and exposure time (Jastrzębska et al., 2017). Chen et al. (Chen et al., 2017) cultured preosteoblasts (MC3T3-E1 and CRL-2593 cells) in MAX phase solutions (Ti_3_AlC_2_, Ti_3_SiC, and Ti_2_AlN) solutions, and these materials showed that they exhibited no toxicity to preosteoblasts and the cells all had active proliferation, with Ti_2_AlN showing the best performance in comparison. Scheibe et al. (Scheibe et al., 2019) explored the cytotoxicity of several commonly used MXene (Ti_3_C_2_, Ti_2_C, and Ti_2_N), exposing human fibroblasts (MSU_1.1_) to MXene for 48 h at concentrations ranging from 10 to 400 μg/mL, and cell viability was consistently above 80% in MSU_1.1_ cells. Jastrzebska et al. (Jastrzębska et al., 2017) exposed normal cell lines (MRC-5, HaCaT) to increasing concentrations (0–500 mg/L) of MXene (Ti_3_C_2_) solution for 24 h. Cell viability declined as the Ti_3_C_2_ concentration increased, and HaCaT was consistently >70% throughout the concentration range tested (0–500 mg/L), and MRC-5 was >70% in the concentration range 0–250 mg/L (Jastrzębska et al., 2017). Aleksandra et al. (Szuplewska et al., 2019a) found that HaCaT, MCF-10A consistently maintained good activity (>70%) even after incubation (incubate) at high concentrations of MXene (Ti_2_CT_x_) (500 μg/mL) for 24 h. However, it should be noted that cell viability significantly decreased after 48 h of exposure (0–500 μg/mL), which may be attributed to the time-dependent toxicity of Ti_2_CT_x_, but HaCaT viability was still >80% in the concentration range (0–62.5 μg/mL). Experiments by Szuplewska et al. (Szuplewska et al., 2019b) also confirmed that after incubation of HaCaT and MCF-10A in Ti_2_NT_x_ solution (0–500 μg/mL) for 24 h, the cell viability treatments showed a slight impairment with increasing concentration. Still, the cell viability was >70% throughout the concentration range. The above studies suggest that MXene has strong cell affinity and good biocompatibility and has no significant effect on cell viability. In contrast, MXene has a greater cytotoxic effect on cancer cell lines (e.g., A375, A549 and MCF-7) than on normal cells (e.g., MRC-5, MCF-10A and HaCaT), which may be related to the differences in cellular metabolism and the chemical composition of the cellular membrane (Jastrzębska et al., 2017; Szuplewska et al., 2019a; Szuplewska et al., 2019b; Scheibe et al., 2019). The generation of oxidative stress in the cell is thought to be a significant contributor to nanoparticle-induced cytotoxicity. The probable reason for the enhanced cytotoxicity of Xene in cancer cells may be due to altered mechanisms of subcellular internalization and the increased production of reactive oxygen species (ROS) due to the oxidative stress phenomenon (Yuan et al., 2018; Gurunathan et al., 2019). Wang et al. (Wang et al., 2020b) designed a three-dimensional crosslinked structured bio-composite membrane (SF@MXene) with flexibility by combining filipin protein and Ti_3_C_2_T_x_. And the composite material showed a survival rate of 99.5% ± 0.8% in HSAS1 cells after 6d of incubation, demonstrating good biocompatibility. This indicates that making full use of the numerous surface functional groups of MXene for modification can help to improve its biological properties further and promote its application in biomedical fields.

In *vivo* toxicity tests, the toxicity is dependent on the dose, duration of treatment, and route of administration. Lin et al. (Lin et al., 2017a) injected 2.0 mg/kg of Nb_2_C-PVP intravenously into mouse and after 28 days, tissue sections and staining of major organs did not show significant acute, chronic pathologic toxicity or adverse effects, and hematological indicators and biochemical parameters also supported this discovery. Li et al. (Li et al., 2021) used MXene (Bi_2_S_3_/Ti_3_C_2_T_x_) in an *in vivo* wound healing assay, and on day 2, a lot of inflammatory cells displayed in the control, Ti_3_C_2_T_x_, and Bi_2_S_3_ groups, except for Bi_2_S_3_/Ti_3_C_2_T_x_. On day 12, some inflammatory cells were still present in the control and Ti_3_C_2_T_x_ groups, suggesting that Bi_2_S_3_/Ti_3_C_2_Tx promotes wound healing. Zhang et al. (Zhang et al., 2019) applied MXene (Ti_3_C_2_T_x_) films to a rat cranial defect model and reconstructed the images with Micro-CT scanning, which revealed that new bone was formed at the defect site and that the new bone extended from the edge of the defect to form bone islands under the MXene films. Nasrallah et al. (Nasrallah et al., 2018) conducted an ecotoxicological evaluation using the zebrafish embryo model and no significant teratogenic effects on zebrafish embryos were observed at 100 mg/mL of Ti_3_C_2_T_x_. Further neurotoxicity and locomotor tests were carried out and toxic effects were found to be insignificant, with no significant effects on neuromuscular activity at 50 μg/mL of MXene. Up now, although long-term toxicity and biosafety have been investigated in lower animal models (e.g., zebrafish or mouse) *in vitro* and *in vivo*, further exploration in higher mammals (e.g., dogs, rabbits, and monkeys) is still needed.

### 3.2 Biodegradability

The bio-degradability of nanomaterials is important to ensure their safety for biological tissues, and evaluating the biodegradability of 2D materials is the basis for translating them into future clinical applications (Zhou et al., 2020). Studies have shown that MXene is degradable. Lin et al. (Lin et al., 2017a) used human myeloperoxidase (hMPO) to simulate the degradation of Nb_2_C *in vivo* and demonstrated the decomposition process by observing its shape change through TEM. Twenty-4 hours after adding the hydroxylase group, the 2D planar structure of the original nanosheets completely disappeared. Upon the addition of hMPO and hydroxylase, MXene was almost completely degraded. Feng et al. (Feng et al., 2019) evaluated the degradation behavior of Mo_2_C by subjecting MXene (Mo_2_C) to PBS and FBS solutions with different pH values by monitoring the changes in UV-vis-NIR absorption spectra of the solutions. The results showed that Mo_2_C had a significant pH-dependent degradation effect, with rapid degradation under alkaline conditions (pH 9.4 and 11.4), as well as gradual biodegradation in neutral PBS solution or medium containing 10% FBS (pH 7.4), while it was relatively stable in acidic environments (pH 3.4, 5.4, and 6.4). Mo_2_C could be degraded in the presence of water and aqueous dissolved oxygen. And the addition of H_2_O_2_ accelerated the degradation process into soluble and harmless MoO_4_^2−^. Han et al. (Han et al., 2018a) injected Ti_3_C_2_-SP intravenously into mice to study its metabolic pathway and observed that Ti_3_C_2_-SP could be gradually excreted in urine and feces after 48 h. The amounts of Ti_3_C_2_-SP excreted were 18.7% and 10.35%, respectively, which indicated that it was biodegradable. Zhang et al. (Zhang et al., 2019) implanted MXene (Ti_3_C_2_) films into rat subcutaneous tissues, and HE staining showed a slight inflammatory reaction after 14d. Granulation tissue was formed around the ruptured MXene, accompanied by a large number of fibroblasts and capillaries, and macrophages adhered to the MXene without significant inflammatory cell infiltration or necrosis, indicating superior biodegradability. The degradation of MXene decreases the toxicity caused by the accumulation of materials in the body, which represents a basis for developing biological applications. However, there are still few studies on the biodegradability of MXene, and how to achieve maximum efficacy while maintaining a safe dosage will be a significant barrier to the further advancement in biomedical.

## 4 Antimicrobial mechanism of MXene

2D nanomaterials have received widespread attention because of their excellent antimicrobial properties. However, research on the antimicrobial mechanisms of MXene began only a few years ago, and a deep understanding of their antimicrobial mechanisms has not yet been established as of today. To the best of our knowledge, a series of pioneering studies have been conducted to unravel these mechanisms. In general, they can be summarized as follows (Turner et al., 2019): physical damage to bacterial membranes due to the sharp edges of the material (Pino et al., 2023); chemical damage by inducing oxidative stress (Gao et al., 2021); synergistic enhancement of antimicrobials by near-infrared phototherapy. The antimicrobial mechanisms of physical and chemical damage of MXene attenuate the possibility of bacteria to be drug-resistant.

### 4.1 Disruption of cell membrane integrity

**FIGURE 3:**
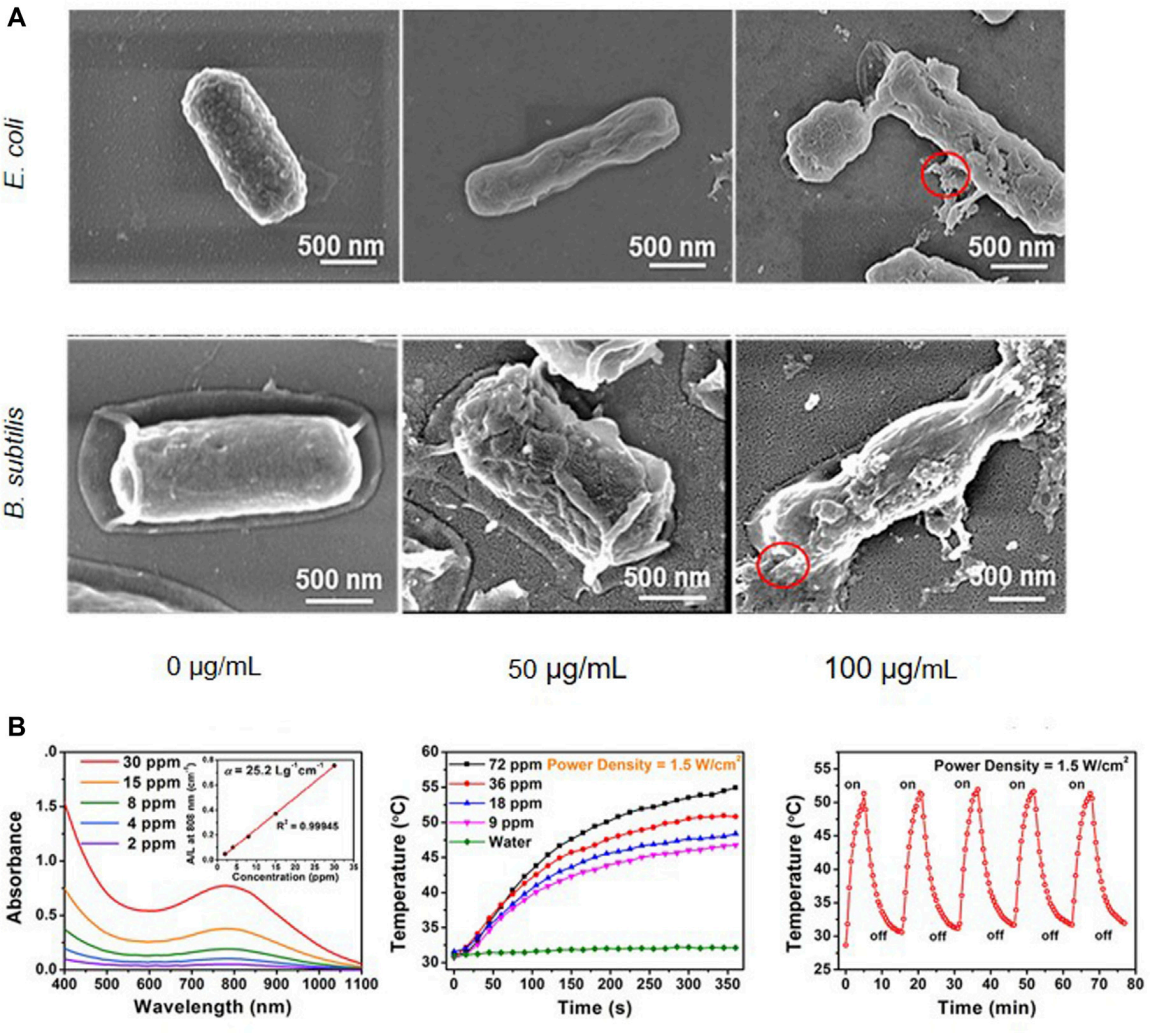
**(A)** At 50 and 100 μg/mL of MXene, both bacteria suffered from prevalent cell lysis indicated by severe membrane disruption and cytoplasmic leakage (see the red circles) (Rasool et al., 2016). Adapted with permission from Kashif Rasool, Mohamed Helal, Adnan Ali, et al. Antibacterial Activity of Ti_3_C_2_T_x_ MXene. Copyright 2016, American Chemical Society (ACS). This publication is licensed under CC-BY. **(B)** MXene composites showed good photothermal effect (Lin et al., 2017b). Adapted with permission from Han Lin, Xingang Wang, Luodan Yu, et al. Two-Dimensional Ultrathin MXene Ceramic Nanosheets for Photothermal Conversion. Copyright 2017, American Chemical Society (ACS).

Mechanical disruption of bacterial membranes by rough surfaces or sharp edges is one of the chief antibacterial mechanisms of 2D nanomaterials. Rasool et al. (Rasool et al., 2016) first investigated the antibacterial properties of Ti_3_C_2_T_x_ MXene and observed the changes in membrane integrity and bacterial morphology after interaction with *E.coli* and *B. subtilis* through SEM and TEM. SEM images showed that no membrane damage or cell death was observed in the control group, and the bacteria were protected by intact cell membrane, while the experimental group showed membrane damage and cytoplasmic leakage. When the concentration of Ti_3_C_2_T_x_ was increased to 100 μg/mL, the bacteria all suffered from generalized cell lysis, which was manifested by severe membrane rupture and cytoplasmic leakage (Figure 3A). The TEM images showed that the Ti_3_C_2_T_x_ nanosheets were strongly adsorbed around the cells and even penetrated into the cells, and all of the intracellular density of the experimental group of bacteria were reduced, indicating that they lost intracellular material. Pandey et al. (Pandey et al., 2020) used ultrathin sectioning of bacteria followed by TEM analysis. They found that Nb_2_C_2_T_x_ and Nb_4_C_3_T_x_ nanosheets were absorbed around the cell walls of *S. aureus* and *E. coli*. Some of the nanosheets were able to enter into the cell walls, resulting in pore formation and disruption of the cell wall and cytoplasmic membrane, efflux of the cellular contents, and significant deformation and damage of the intracellular structures. Arabi Shamsabadi et al. (Arabi Shamsabadi et al., 2018) measured the changes in the growth of *B. subtilis* and *E. coli* strains induced by Ti_3_C_2_T_x_ nanosheets of different sizes, and the growth kinetics tests showed that the sharp edges of Ti_3_C_2_T_x_ nanosheets caused damage to *B. subtilis* and *E. coli* through direct physical interaction with the bacterial membrane surface, which induced damage to the bacterial envelope, leading to cytoplasmic DNA release. Interestingly, this antimicrobial capacity showed a nanosheet size and exposure time dependence, with nanosheets with smaller sizes being more likely to enter the microorganisms and disrupt the cytoplasmic components through physical penetration or endocytosis (Pandey et al., 2020), in agreement with the findings of Rasool et al. (Rasool et al., 2017). Further LDH release assay was carried out to quantify the extent of cellular damage. LDH release increased significantly with increasing concentrations of Ti_3_C_2_T_x_, indicating that both cell walls and contents were damaged and that the antimicrobial capacity was dose-dependent (Rasool et al., 2016). The aforementioned experiments suggest that the sharp edges of MXene result in physical damage to bacterial membrane as one of its vital antimicrobial mechanisms.

### 4.2 Induced generation of oxidative stress

As we know, oxidative stress is a joint bactericidal mechanism for metal, metal oxide, and carbon-based nanomaterials. MXene induces the onset of oxidative stress by inducing the generation of ROS (^1^O_2_, OH, O^2−^, H_2_O_2_), which causes damage to proteins, lipids, DNA, and RNA (Jastrzębska et al., 2021). MXene typically has a negative zeta potential, which contributes to its affinity to cell surfaces and ease of formation of chemical reactions. In addition, with its excellent conductivity, MXene can induce ROS production through generating electron transfer bridges with the cellular lipid bilayers (Rasool et al., 2016). Pandey et al. (Pandey et al., 2020) used glutathione (GSH) oxidation to detect oxidative stress mediated by Nb_2_CT_x_ and Nb_4_C_3_T_x_ when exploring the antimicrobial mechanism of Nb_2_CT_x_ and Nb_4_C_3_T_x_. Both materials showed GSH depletion, but the Nb_4_C_3_T_x_ group showed higher oxidative stress. The difference in oxidative stress between the two materials may be related to the atomic structure, Nb_4_C_3_ (n = 3) has higher interlayer spacing, greater charge transfer activity and oxidization ability than Nb_2_C (n = 1), in agreement with Jastrzębska et al. (Szuplewska et al., 2019b). Zheng et al. (Zheng et al., 2020) who investigated the effectiveness of the antibacterial properties of MXene, calculated that the ROS produced by the MXene group was 1.8 folds higher than that of the control group, and further performed a lipid peroxidation assay to verify whether the ROS produced caused any damage to the membranes of the bacteria, and found that the MXene group was able to oxidize the bacterial membranes, which was 1.3 folds higher than the control group. This indicates that MXene can induce oxidative stress by generating ROS, which can cause damage to bacterial membranes. However, they did not further explore the kind of ROS produced by MXene. Rasool et al. (Rasool et al., 2016) monitored the amount of O^2−^ produced in different concentrations of Ti_3_C_2_T_x_ by the XTT method, yet no significant uptake was detected, indicating that O^2−^ makes a negligible contribution to the antimicrobial activity of Ti_3_C_2_T_x_. Therefore, a more in-depth study of MXene is necessary to investigate ROS formation other than superoxide anion (OH, ^1^O_2_, H_2_O_2_, etc.).

### 4.3 Near-infrared light-based phototherapy synergized (NIRL)

For antibiotic-resistant and oxidative stress-resistant bacteria, such as methicillin-resistant *Staphylococcus aureus* (MRSA), whose growth is challenging to be inhibited by the antimicrobial action of MXene. NIRL therapies, consisting of photodynamic therapy (PDT) and photothermal therapy (PTT), have provided new strategies to inhibit such bacteria (Ezraty et al., 2017). PTT is an innovative method of combating drug-resistant bacteria by transferring light energy into heat through photothermal agents (PTAs), which are delivered to the bacterial surface, resulting in membrane rupture, protein denaturation and irreversible damage (Zou et al., 2017). PDT, on the other hand, utilizes photosensitizers (PS) to transfer energy to the surrounding oxygen molecules after receiving light, generating ROS. Excessive ROS accumulation will deplete antioxidants and affect the bacterial membranes, leading to membrane rupture (Yuan et al., 2020). Both strategies are highly dependent on the photoactivation properties of the nanomaterials. MXene has excellent light-absorbing properties across the full range of visible light and NIR, demonstrating excellent photoenergy transformation potential. Based on the intrinsic photothermal and photodynamic properties of MXene, it shows promising applications in PTA or PS (Huo et al., 2021; Xiang et al., 2021) (Table 1). The UV-vis-NIR absorption spectra obtained on Ti_3_C_2_T_x_ nanosheets showed unique absorption in the NIR region ranging from 750 to 850 nm, similar to conventional metal nanoparticles showing the localized surface plasmon resonance (LSPR) effect (Lin et al., 2017b). The absorption range of MXene is just within the biological NIR window, making it highly suitable for biomedical applications. The extinction coefficient at 808 nm (25.2 Lg^−1^ cm^-1^) was further measured, which is higher than that of GO nanosheets (3.6 Lg^-1^ cm^−1^) and WS_2_ nanosheets (23.8 Lg^−1^ cm^−1^). The photothermal conversion efficiency of Ti_3_C_2_T_x_ nanosheets was calculated to be 30.6%, higher than that of Au nanorods (AuNRs) (21%), Cu_2-x_ Se NCs (22%), and Cu S_95_ NCs (25.7%). Under 5 laser on/off cycles, there was no significant deterioration in the photothermal performance of Ti_3_C_2_T_x_, indicating its potential as a persistent photothermal agent (Figure 3B). Rosenkranz et al. (Rosenkranz et al., 2021) compared the antimicrobial effects of MXene and MXene combined with PTT; after NIR-PTT, SEM showed massive cellular debris and a significant increase in DNA release, leading to irreversible cell death, whereas bacteria in the MXene alone group showed regrowth after release from the vortexing of the nanomaterials. This suggests that the toxicity of MXene to bacteria will be enhanced by NIR spectroscopic treatment, in agreement with Lu et al. and Zhu et al. (Zhu et al., 2020; Lu et al., 2021). Liu et al. (Liu et al., 2017b) modified Ti_3_C_2_T_x_ by adding Al^3+^, and the photothermal conversion efficiency was enhanced to 58.3%. With the increase of laser power density, the degree of temperature elevation of Ti_3_C_2_T_x_ dispersion increased. After 30 min of irradiation, no significant photobleaching of Ti_3_C_2_T_x_ nanosheets was observed. Even at low concentration (25 μg/mL), the temperature of Ti_3_C_2_T_x_ dispersion temperature reached the plateau rapidly even at low concentration (25 μg/mL), indicating that Ti_3_C_2_T_x_ nanosheets have more excellent photothermal properties and stability. In addition, the MXene-based materials demonstrated an excellent ability to promote ROS generation in the study. The production of ^1^O_2_ in the MXene group was detected under near-infrared light irradiation with 1,3-diphenylisobenzofuran (DPBF), and the production of ^1^O_2_ was attributed to the energy transfer of photoexcited electrons to triplet oxygen (^3^O_2_) from Ti_3_C_2_T_x_ as well as the LSPR effect of metal nanoparticles (Han et al., 2018a; Zhang et al., 2019). Another study also confirmed that after irradiation with a 532 nm laser (20 mW) for 30 min, the MXene group showed a decrease in absorbance at 417 nm, which produced a single linear state of oxygen (Liu et al., 2023a). MXene produces a large amount of ROS from the material’s PDT under near-infrared light irradiation, while the material’s superior PTT raises the ambient temperature of the bacteria. High temperatures cause irreversible bacterial damage by denaturing bacterial proteins and increasing the permeability of the bacterial membrane, which allows ^1^O_2_ produced during photocatalytic process to penetrate into bacteria easier, leading to death by damaging bacterial membranes, proteins and DNA (Wu et al., 2021a). In summary, MXene is considered a very promising PTA and PS.

**TABLE 1:** Photothermal conversion efficiency of some MXene.

Mxene	Photothermal conversion efficiency	References
Ti_3_C_2_T_x_	30.60%	Lin et al. (2017b)
Ag/Ti_3_C_2_T_x_	49.9%	Zhu et al. (2020)
Modified Ti_3_C_2_T_x_	58.3%	Liu et al. (2017b)
Bi_2_S_3_/Ti_3_C_2_T_x_	35.43%	Li et al. (2021)
Ag_2_S/Ti_3_C_2_	27.80%	Wu et al. (2021a)
LPFEG-Mxene	40.79%	Riaz et al. (2023)
Ti_3_C_2_@PDA	42.3%	Li et al. (2022c)
GdW_10_@ Ti_3_C_2_	21.9%	Zong et al. (2018)
MXene@Fe_3_O_4_/Au/PDA	48.70%	Liu et al. (2022)
MXene@Ag@PDA	43.80%	Liu et al. (2023b)
MXene@AgAu@PDA	66.60%	Liu et al. (2023b)
Nb_2_C	28.6%	Han et al. (2018b)
MnO_x_/Ta_4_C_3_	34.9%	Dai et al. (2017)

## 5 Application of MXene in the antimicrobial field

**FIGURE 4:**
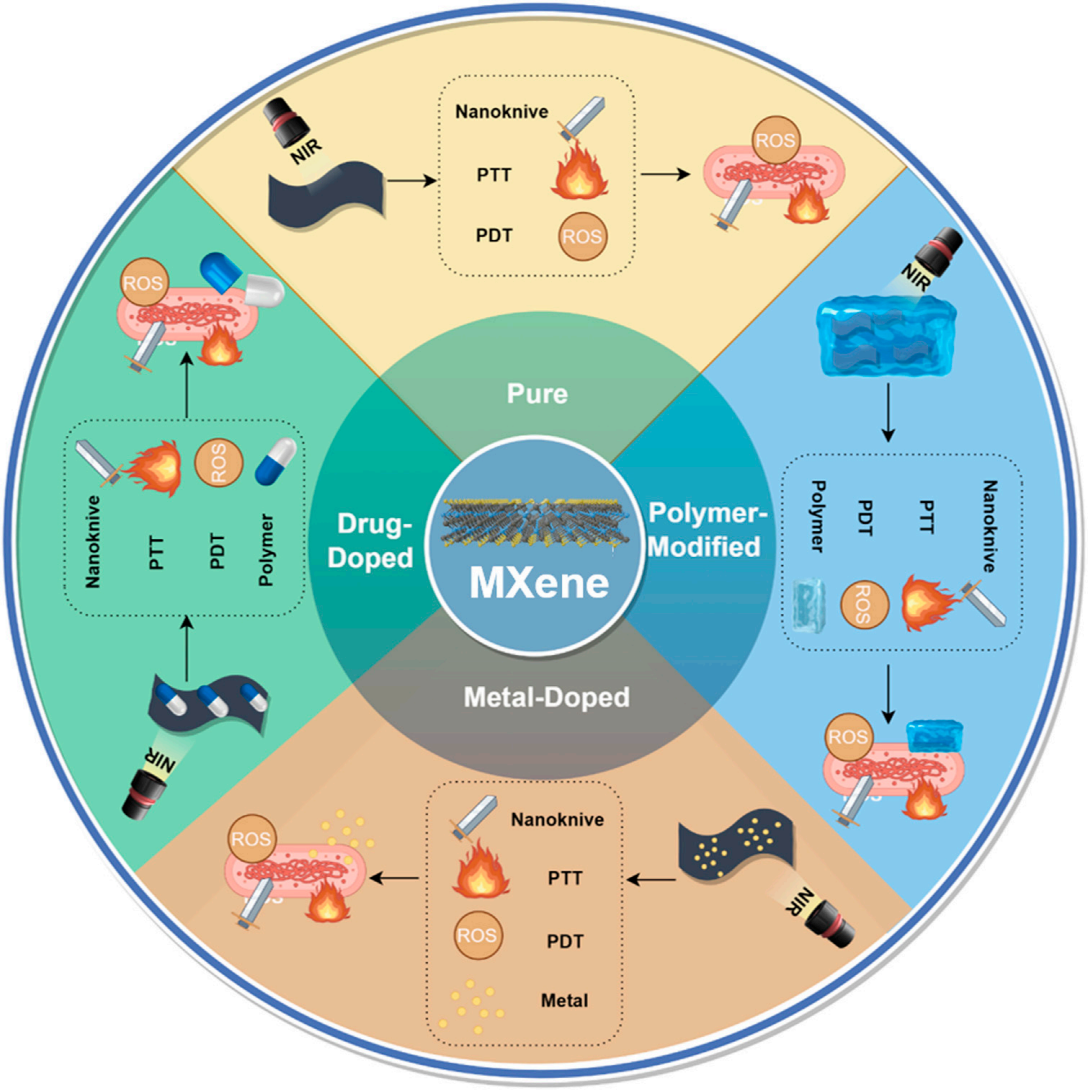
The application of MXene and the main antibacterial strategies of pure MXene, polymer-modified MXene, metal-doped MXene and drug-doped MXene.

Antimicrobial drug resistance is one of the biggest threats to worldwide health. With the aim of tackling this challenge, researchers have suggested the creation of hybrid antimicrobial materials to prevent the emergence of drug resistance by simultaneously targeting bacteria via a multitude of mechanisms (Razzaque, 2021; Iravani et al., 2022). MXene has shown attractive antimicrobial potential by its unique properties. In this paper, an attempt has been made to categorize the materials interacting with MXene into polymers, pharmaceuticals, metallic nanomaterial, and to state its applications in antimicrobial field according to this categorization. (Figure 4). Finally, we summarized the application of MXene in antibacterial (Table 2).

**TABLE 2:** Summary of the application of MXene in antibacterial.

Mxene	Etching	Strategies	Bacterial strains	Laser	Application	References
Ti_3_C_2_T_x_	LiF/HCl	Nano-knife and oxidative stress reactive	*E. coli* and *B. subtilis*	—	Antibacterial film or coating	Rasool et al. (2016)
Nb_2_CT_x_ and Nb_4_C_3_T_x_	HF	Nano-knife and oxidative stress reactive	*E. coli* and *S. aureus*	—	Bacteriostatic applications	Pandey et al. (2020)
Ti_3_C_2_T_x_	LiF/HCl	Nano-knife and oxidative stress reactive	*E. coli* and *B. subtilis*	—	Antibacterial film for sewage treatment	Rasool et al. (2017)
Ti_3_C_2_T_x_	LiF/HCl	Nano-knife	*E. coli* and *S. aureus*	—	Skin infection wound healing	Mayerberger et al. (2018)
Bi_2_S_3_/Ti_3_C_2_T_x_	HF	PDT and PTT and oxidative stress reactive	*E. coli* and *S. aureus*	0.7 W/cm^2^, 10 min	Infection wound healing	Li et al. (2021)
MXene-AuNCs	LiF/HCl	Nano-knife and oxidative stress reactive and ability of Ag+	*E. coli* and *S. aureus*	—	Bacterial infection treatment and defend against the biofilm formation	Zheng et al. (2020)
Ag/Ti_3_C_2_T_x_	HF	PTT	*E. coli* and *B. subtilis*	1.5 W/cm^2^, 15 min	Infection wound healing	Zhu et al. (2020)
Ru/Ti_3_C_2_T_x_	LiF/HCl	Nano-knife and PTT and oxidative stress reactive	*E. coli* and *S. aureus*	0.15 W/cm^2^, 30 min	Bacterial infection treatment	Liu et al. (2023a)
LPFEG-Mxene	LiF/HCl	Nano-knife and PTT	*E. coli* and *S. aureus* and *P. aeruginosa*	2.0 W/cm^2^, 5 min	Bacterial infection treatment and wound healing	Riaz et al. (2023)
Ti_3_C_2_@PDA	HF	PTT and the ability of HbO_2_ and H_2_O_2_	*E. coli* and *S. aureus*	1.0 W/cm^2^, 10 min	Infected diabetic wound healing	Li et al. (2022c)
MXene@Fe_3_O_4_/Au/PDA	LiF/HCl	Nano-knife and magnetolytic-photothermal coupling antibacterial	*E. coli* and *S. aureus*	2.0 W/cm^2^, 6 min	Antibacterial in chemical industry and environmental treatment	Liu et al. (2022)
MXene@AgAu@PDA	LiF/HCl	PPT and the ability of Au and Ag	*E. coli* and *S. aureus*	2.0 W/cm^2^, 6 min	Antibacterial in water pollution treatment	Liu et al. (2023b)
SCFPEEK-PDA-Ti_3_C_2_T_x_	LiF/HCl	PTT	*E. coli* and *S. aureus*	1.0 W/cm^2^, 10 min	Orthopedic internal fixation and implant materials	Du et al. (2022)
MX-CS	LiF/HCl	Nano-knife and PTT	MRSA	1.6 W/cm^2^, 5 min	Treatment of MRSA-infected diseases	Dong et al. (2023)
GelMA/β-TCP/Alg/MXene	LiF/HCl	Nano-knife and PTT and oxidative stress reactive	*E. coli* and *S. aureus*	1.5 W/cm^2^,10min	Individualized treatment of infected bone defects	Nie et al. (2022)
MXene/PVA	LiF/HCl	PPT	*E. coli* and *S. aureus*	1.5 W/cm^2^, 10 min	Infected wound healing	Li et al. (2022b)
MXene-PEIS	HF	Nano-knife and the ability of quaternary ammonium	*E. coli* and *S. aureus*	—	Anti-bacterial and anti-biofouling coating	Zeng et al. (2023)
MTX/Ag	LiF/HCl	Nano-knife and PTT and PDT and oxidative stress reactive and ability of Ag^+^	*E. coli* and *S. aureus*	2.0 W/cm^2^, 30 min	Mildew resistant coating	Qin et al. (2023)
Cu_2_O/MXene	HF	Nano-knife and oxidative stress reactive and surface plasmon resonance (SPR) and the ability of Cu^2+^	*S. aureus* and *P. aeruginosa*	—	Bacteriostatic applications	Wang et al. (2020c)
ZnTCPP/Ti3C2T_x_	HF	PDA and oxidative stress reactive	*E. coli* and *S. aureus*	—	Bacterial infection treatment and wound healing	Cheng et al. (2022)
MXene@CeO_2_	LiF/HCl	Nano-knife and oxidative stress reactive	*E. coli* and *S. aureus* and MRSA	—	Bacterial infection treatment and wound healing	Zheng et al. (2021)
MXene/Zinc	LiF/HCl	Nano-knife and PTT and oxidative stress reactive and ability of Zn^2+^	*E. coli* and *S. aureus*	1.5 W/cm^2^, 10 min	Bacterial infection treatment and wound healing	Hu et al. (2024)
Cip-Ti_3_C_2_	LiF/HCl	Nano-knife and PTT	MRSA	1.0 W/cm^2^, 15 min	Resistant bacterial infection treatment and wound healing	Zheng et al. (2022)
d-Ti_3_C_2_T_x_	HF	Nano-knife	Trichoderma	—	Antifungal application	Lim et al. (2020)
AgNP/MXene	LiF/HCl	Nano-knife and ability of Ag^+^	*E. coli*	—	Antibacterial film for sewage treatment and water purification	Pandey et al. (2018)
Ti_3_C_2_T_x_	HF	Nano-knife and PTT	*E. coli, K. pneumoniae, P. aeruginosa, A. baumannii, S. typhi, Shigella, Burkholderia cepacia, Enterobacter cloacae, Enterobacter aerogenes, P. mirabilis, S. aureus, VRE, Enterococcus faecalis, Streptococcus agalactis, B. subtilis*	0.4 W/cm^2^, 20 min	Eradicate resistant bacteria and biofilms	Wu et al. (2021b)
Ti_3_C_2_T_x_	HF	Nano-knife and oxidative stress reactive	*E. coli*	—	Photocatalytic and bacteriostatic applications	Rajavel et al. (2021)
Ti_3_C_2_T_x_	LiF/HCl	Nano-knife and oxidative stress reactive	*E. coli* and *S. aureus*	—	Solar-Driven Water Purification	Zha et al. (2019)
Ti_3_C_2_/CoNWs	LiF/HCl	PDT and PTT and oxidative stress reactive	*E. coli* and *S. aureus*	1.5 W/cm^2^, 20 min	Antibacterial coatings on an orthopedic implant	Liu et al. (2021)
M-HAS	HF	Nano-knife and PDT and oxidative stress reactive and ability of Ag^+^	*S. aureus*	0.8 W, 30 min	Photocatalytic and bacteriostatic applications	Lv et al. (2021)
C-T@Ti_3_C_2_	HF	Oxidative stress reactive and SDT	*E. coli* and *S. aureus* and MRSA	—	Resistant bacterial infection treatment and promote bone tissue regeneration	Yu et al. (2023)
PVA/PDA/MXene/CuS	NaF/HCl	PPT and PDT and oxidative stress reactive and ability of Cu^2+^	*E. coli* and *S. aureus*	1.4 W/cm^2^, 30 min	Infected wound healing	Su et al. (2023)
MZ-8/PLA	HF	PPD and PPT	*E. coli* and MRSA	1.0 W/cm^2^, 5 min	Treatment of MRSA-infected diseases	Zhang et al. (2021)
MXene/PDA/Ni^2+^	LiF/HCl	Nano-knife	*E. coli*	—	Antibacterial coating for wearable electronics	Gong et al. (2021)

### 5.1 Pure MXene

Pure MXene can be used in antimicrobial applications in the form of coatings. Huang et al. (Huang et al., 2022) prepared Ti_3_C_2_T_x_ coating on the surface of plain titanium by anodic electrophoretic deposition. The experimental data shows that the coating has great hydrophilicity and bonding strength, which can inhibit the adhesion of *S. aureus* and MRSA and hinder biomembrane formation, effectively preventing the peri-implantitis. Rasool (Rasool et al., 2017) et al. loaded Ti_3_C_2_T_x_ as a surface coating material onto polyvinylidene fluoride (PVDF) membranes to investigate its potential in wastewater purification. The results showed that Ti_3_C_2_T_x_ significantly improved the hydrophilicity of the membranes and the growth inhibition of *B. subtilis* and *E. coli* by Ti_3_C_2_T_x_/PVDF membranes reached 73% and 67%, respectively. There was no bacterial growth in the permeate through Ti_3_C_2_T_x_/PVDF membranes. The impact of environmental conditions on membrane efficiency is also an important factor. More than 99% inhibition of *E. coli* and *B. subtilis* was observed for aged membranes compared to fresh membranes, which provides new ways for the development of efficient antimicrobial membranes for wastewater processing. Diedkova et al. (Diedkova et al., 2023) deposited MXene coatings onto Polycaprolactone (PCL) nanofibers. They observed bacterial adhesion and biofilm formation, which showed that the MXene coatings reduced bacterial adhesion to the PCL membranes and suppressed the colonization of *E. coli* and *P. aeruginosa*. In order to improve the antimicrobial and osseointegration capabilities of orthopedic implants, Du et al. (Du et al., 2022) doped Ti_3_C_2_T_x_ in polyetheretherketone (PEEK), and the incorporation of Ti_3_C_2_T_x_ endowed the composites with bimodal therapeutic functions of photothermal sterilization and *in vivo* osseointegration, with a 100% sterilization rate under near-infrared light irradiation. Yang et al. (Yang et al., 2021) prepared Nb_2_C@TP by doping Nb_2_C onto titanium plates, and the results showed that under the near infrared light irradiation, not only could it destroy the already formed biofilm, but also the thermal damage impaired the bacterial motility system, further preventing biofilm formation and diffusion, with an antimicrobial rate of 99%. At the same time, Nb_2_C@TP can also reduce excessive inflammatory response and ROS production, and promote angiogenesis and tissue regeneration, bringing hope for medical implant-related infection cases. Therefore, MXene is considered a promising biomedical material.

In summary, pure MXene can be prepared as coatings or films, and thanks to its excellent hydrophilicity, loading in bioimplant materials can effectively improve the antibacterial ability of implants by inhibiting bacterial adhesion and biofilm formation. Therefore, MXene is considered to be a potential biomedical material.

### 5.2 Polymer-modified MXene

The modification of polymers is to be helpful in overcoming the shortcomings present in the material itself, neutralizing the charge on the surface of the nanoparticles, and preventing nanoparticle aggregation (Lukyanov and Torchilin, 2004).MXene is characterized by spontaneous aggregation and uncontrollable protein corona formation in physiological environments, which greatly limits its photothermal effects and practical applications. Dong et al. (Dong et al., 2023) prepared MX-CS hydrogels by mixing Ti_3_C_2_T_x_ suspension and acidic Chitosan (CS) solution, which allowed MRSA cells and MXene-induced high-temperature aggregation around the hydrogel, thus improving the photothermal effect and enhancing the photothermal anti-MRSA activity (>99%). Zhou et al. (Zhou et al., 2021) developed biodegradable HPEM scaffolds by crosslinking branched MXene@PDA, polyglycerol-ethyleneimine (PGE), and hyaluronic acid oxide (HCHO). The incorporation of MXene@PDA improved the thermal stability and electrical conductivity of the scaffolds. The HPEM scaffolds can significantly accelerate wound healing and skin reconstruction in MRSA-infected wounds through effective anti-infection (99.03% efficiency of inactivation of MRSA), promoting the process of cell proliferation and angiogenesis, and stimulating the formation of granulation tissue. Nie et al. (Nie et al., 2022) mixed Ti_3_C_2_T_x_ with gelatin methacrylate (GelMA), sodium alginate (Alg), and *β*-TCP to make bio-ink and prepared composite hydrogel scaffolds by 3D printing, which not only inhibited the growth of bacteria but also killed the bacteria that had already adhered to the scaffolds and proliferated in large quantities under NIR irradiation. The bactericidal rate reached 98%, which can effectively treat infected bone defects in the mandible, and the scaffolds also showed sustained anti-infective ability as the scaffolds degraded and MXene was slowly released. Inspired by the soft and tough properties of muscles and ligaments, Li et al. (Li et al., 2022b) synthesized MXene@PVA hydrogels by targeted freezing-assisted salting out, which possessed high water content, high swelling, and hydrophilicity, good mechanical properties, and biocompatibility, as well as good photothermal conversion properties. *In vitro* antimicrobial tests further showed that MXene@PVA hydrogel inhibited *E. coli* and *S. aureus* by 98.3% and 95.5%, respectively. Thanks to the electrical conductivity of MXene, the hydrogel could establish a cellular communication network by enhancing the electrical signals, up-regulating the expression of relevant genes, and promoting the proliferation of the cells, which could significantly promote infected wounds healing. Zhang et al. (Zhang et al., 2023) introduced Ti_3_C_2_T_x_ into the interpenetrating polymer network (IPN) constructed by GA-modified collagen (GCol) and poly (acrylic acid) and prepared GCol-MX-PAA multifunctional hydrogels by *in situ* polymerization, optimizing the adhesion properties and biocompatibility of the hydrogels. The adhesion, biocompatibility, and mechanical properties of the hydrogel were optimized, and the bacterial inhibition rate was greater than 95% under the synergistic effect of PTT, which had an excellent antimicrobial effect.

MXene is easily dislodged in wounds, and direct contact with tissues may result in the persistence of MXene nanosheets in tissues, causing secondary damage injury (Ding et al., 2023). Loading MXene into nanofibers helps to prevent MXene nanosheets from directly contacting the tissue. Mayerberger et al. (Mayerberger et al., 2018) functionalized electrostatically spun CS nanofibers with Ti_3_C_2_T_x_ to develop a flexible bandage material, a dressing that can cause mechanical damage to bacterial membranes, kills 95% of *E.coli* bacteria, and can be used as an excellent wound dressing material.

Bacterial infections and oxidative damage caused by various reactive oxygen species (ROS) pose a major threat to human health by impeding wound healing. Riaz (Riaz et al., 2023) et al. co-assembled Mxene with LPFEG and synthesized a multifunctional chiral supramolecular composite hydrogel system, LPFEG-Mxene, which exhibited broad-spectrum ROS-scavenging antioxidant capacity and significant photothermal antioxidant activity against *E.coli*, *P. aeruginosa,* and *S. aureus* showed significant photothermal antimicrobial activity, showing great potential in antimicrobial coatings and healing of infected wounds. Li et al. (Li et al., 2022c) prepared a hydrogel for diabetic wound healing using hyaluronic acid-graft-dopamine (HA-DA) and polydopamine (PDA) encapsulated with Ti_3_C_2_T_x_ and oxyhemoglobin/hydrogen (HbO_2_/H_2_O_2_). MXene converts NIR to heat, kills bacteria, and scavenges ROS to maintain intracellular redox homeostasis and eliminate oxidative stress. The PDA coating further strengthens the antimicrobial and anti-oxidant properties of MXene while promoting cross-linking of the nanosheets into the hydrogel, and the incorporation of HbO_2_ also endowed the dressing with the ability to deliver oxygen. The infected diabetic wound healing was significantly accelerated by oxygenation, eliminating ROS, eradicating bacteria, and promoting angiogenesis.

Wound healing and skin regeneration in drug-resistant bacterial infections is a challenge, and conventional dressings are primarily involved in the passive healing procedure and seldom participate in active wound healing through stimulation of skin cell behavior. Mao et al. (Mao et al., 2020) reported a study using electrical stimulation to accelerate skin wound closure. An rBC/MXene hydrogel was prepared by mixing Ti_3_C_2_T_x_ and bacterial cellulose (BC), which significantly improved the conductivity of the hydrogel. Compared with the rBC hydrogel alone, the rBC/MXene hydrogel could promote skin wound healing. *In vivo* and *in vitro* data showed that the hydrogel could further accelerate the wound healing process under the synergistic effect of electrical stimulation (ES). Blending ES with hydrogel therapy provides an effective management strategy to accelerate wound closure.

Polymers with antimicrobial properties are found in nature, such as quaternary ammonium salts, natural cationic antimicrobial polymers, etc. Inspired by various functional approaches for antifouling surfaces, Zeng et al. (Zeng et al., 2023) self-assembled a polydopamine layer onto the surface of MXene and then introduced a quaternate polyethyleneimine derivative (PEIS) to the PDA layer by Michael addition to acquire amphiphilic ionic polymer-functionalized (MXene-PEIS). The prepared MXene-PEIS nanosheets exhibited remarkable antibacterial and anti-biofouling properties (75% and 88% inhibition against *E. coli* and *S. aureus*, respectively), which was contributed by the synergistic effect of MXene and quaternary ammonium groups on the antimicrobial its.

In summary, the introduction of polymers has been observed to address some of its limitations, including its propensity to aggregate and form unmanageable protein corona in physiological settings, as well as being prone to detachment in wound sites. Additionally, the stability of MXene is enhanced by the introduction of polymers, which helps to prolong its efficacy. Moreover, the use of polymers with antimicrobial properties can lead to a synergistic effect, augmenting the antimicrobial characteristics of the materials and broadening the scope of MXene’s potential applications.

### 5.3 Metal-doped MXene

Metal and metal oxide nanoparticles (e.g., Cu, Ag, Zn, etc.) possess excellent antimicrobial activity and are widely used in various applications. The antimicrobial activity of metal and metal oxide nanoparticles is associated with bacterial death through oxidative stress, protein dysfunction, and membrane damage (Lemire et al., 2013; Shifrina et al., 2020).

Silver nanoparticles (Ag NPs) have shown excellent antimicrobial properties against a wide range of microorganisms, but Ag^+^ is unstable in the environment, and high doses of Ag have potential cytotoxicity and possible *in vivo* retention, which limits its use in biomedical applications (Qing et al., 2018; Dedman et al., 2020). Therefore, scholars have been trying to find a good carrier to control the release of Ag^+^ and reduce the toxicity. Qin et al. (Qin et al., 2023) prepared MTX/Ag by ultrasonically-impelled intercalation of MXene into montmorillonite and further *in situ* reduction and immobilization of Ag NPs. The results show that the micro-nano-protective structure formed by MXene embedded in MMT can immobilize Ag well and prevent the toxic reaction caused by Ag leakage, and the photothermal effect of NIR stimulation of MXene can control the release of Ag. Due to the release of reactive oxygen species and PTT, as well as Ag^+^, MTX/Ag exhibited excellent and long-lasting antimicrobial activity (inhibition rate >99%). In addition, when MTX/Ag was mixed with eucalyptus powder and placed in a humid environment, the control group developed mold spots on the third day, while the MTX/Ag-NIR group was not infected by mold at all. Due to the spillage of Ag, the filter paper, which was in direct contact with the wood powder, also did not show infection, providing a new direction for the anti-mold coating of wood. Ag_2_S is an excellent PTT material, but the narrow-forbidden band of 0.9 eV accelerates the complexation of photogenerated electrons and holes in Ag_2_S, which is unfavorable to the photocatalytic reaction, thus limiting its application in biomedical fields. To overcome this difficulty, Wu et al. (Wu et al., 2021a) introduced Ti_3_C_2_T_x_ and prepared Ag_2_S/Ti_3_C_2_. The introduction of Ti_3_C_2_ improved the separating efficiency of the photogenerated charge carriers of the composite enhanced the photo-catalytic activity and ROS generation. The synergistic inhibition rate of PTT and PDT of Ag_2_S/Ti_3_C_2_ was up to 99.99%, effectively promoted the infected wounds healing in mouse.

Conventional AuNPs are inert to bacteria, and when their core size is less than 2 nm, the ultra-small gold nanoclusters AuNCs can be internalized into the bacteria to cause the accumulation of ROS, which disrupts the metabolism and leads to the death of bacteria, showing potent antibacterial activity (Zheng et al., 2017). Zheng et al. (Zheng et al., 2020) coupled AuNCs to the surface of MXene nanosheets for the first time, and the synergistic antibacterial ability was achieved (antibacterial rate >98%). Sharp MXene nanosheets can penetrate bacterial membranes. In addition, the formation of biofilms was effectively inhibited by the construction of crumpled MXene-AuNCs structures. The hydrophobic surface of the crumpled structures prevents bacterial adhesion, as well as the crumpled structure may contain a higher density of biocides to enhance bactericidal effect.

Cuprous oxide (Cu_2_O) is a potentially inexpensive biocide with a wide range of applications in the antimicrobial field. However, severe photocorrosion shortens the time of Cu_2_O-induced ROS generation because of the overaccumulation of photogenerated electrons and holes within Cu_2_O crystals. In addition, the released Cu^2+^ destroys the Cu_2_O semiconductor structure, resulting in the inability of Cu_2_O to generate ROS. Therefore, there is an urgent need to add effective catalysts to the sterilization system of Cu_2_O rationally. Wang et al. (Wang et al., 2020c) designed a stable Cu_2_O-anchored MXene nanosheets, Cu_2_O/MXene, using MXene as a conductor and Cu_2_O as a semiconductor. The results show that the incorporation of MXene not only improves the separation efficiency of electron-hole pair Cu_2_O but also effectively enhances the electric field |E| and generates more ROS to kill bacteria through the LSPR phenomenon. In addition, Cu and Cu^2+^ can also produce toxic effects on bacteria due to denaturation of bacterial DNA. Under the synergistic effect of Cu and MXene, the bacterial inhibition efficiencies of Cu_2_O/MXene against *S. aureus* and *P. aeruginosa* reached 95.59%and 97.04%, respectively, which were significantly greater than those of MXene and Cu_2_O.

When exposed to near-infrared light, light-responsive materials produce thermotherapy and ROS to kill bacteria quickly. However, in practice, it would be more convenient and cheaper if these effects could be triggered by visible light, especially on wounds (Xu et al., 2021a). Porphyrins are widely used as photocatalysts or photosensitizers due to their strong visible light trapping ability, low photogenerated electron-hole complexation, and fast transfer of photogenerated carriers. Cheng et al. (Cheng et al., 2022) prepared ZnTCPP/Ti_3_C_2_T_x_ by hydrothermal method, and under visible light irradiation, ROS generation was observed in the ZnTCPP/Ti_3_C_2_T_x_ group, while it was almost absent in the Ti_3_C_2_T_x_ group. *In vitro*, antimicrobial experiments showed no significant change in bacterial viability under a dark environment. After 10 min of visible light irradiation, the inhibition rates of ZnTCPP/Ti_3_C_2_T_x_ against *S. aureus* and *E. coli* reached 99.86% and 99.92%, respectively, which were significantly higher than those of the Ti_3_C_2_T_x_ group. Moreover, the results also indicated that ZnTCPP/Ti_3_C_2_T_x_ could promote wound healing due to the enhanced photocatalytic activity.

MXene has a weak ability to scavenge ROS and is unable to remove excess ROS around the wound. The accumulation of ROS causes malignant oxidation and severe inflammatory responses, further impeding wound healing and causing pain in patients. To overcome this deficiency, Zheng et al. (Zheng et al., 2021) doped anti-inflammatory nanoparticles CeO_2_ and Ti_3_C_2_T_x_ into dynamic chemically cross-linked hydrogels to prepare injectable multifunctional hydrogel scaffolds (FOM). *In vitro* experiments showed that FOM could protect L929 cells effectively from the wound microenvironment and provide oxygen while alleviating oxidative stress. In addition, FOM exhibited excellent antimicrobial properties, with 100% inhibition of *E. coli*, *S. aureus,* and MRSA. The bacterial suppression and wound healing ability of FOM were further evaluated in the MRSA-infected mouse. FOM plays an important role in eliminating MRSA bacteria, promoting cell proliferation, angiogenesis, and re-epithelialization, and can significantly accelerate the healing of MRSA-infected wounds.

Physical damage to the bacterial membrane induced by the sharp edges of MXene in contact with bacteria is an important antibacterial strategy. However, both MXene and bacterial surfaces are negatively charged, making it difficult for them to contact fully. Zinc (Zn) is involved in many cellular processes and is one of the essential elements in many physiological processes. Zn^2+^ is positively charged and can attract negatively charged bacteria to damage bacterial membranes through electrostatic action (Nimmanon et al., 2021). Hu et al. (Hu et al., 2024) developed a natural polysaccharide hydrogel and embedded Ti_3_C_2_T_x_ and Zn^2+^ in it. The embedding of Zn^2+^ enhanced the electrostatic binding between the hydrogel and the negatively charged bacteria, which facilitated the role of physical antimicrobial mechanism, shortened the thermal conductivity distance of the PTT, and improved the photo-thermal conversion efficiency. While, MXene produces physical damage to the cell membrane, promoting the entry of Zn^2+^ into the bacteria and exerts an antibacterial effect. The integration of Zn^2+^ and MXene realizes effective antimicrobial properties and promotes wound healing and skin regeneration.

Researchers have discovered that the combination of MXene and positively charged metal ions can effectively bind to bacterial surfaces through electrostatic forces. This binding process results in physical harm to the bacteria, the production of ROS, and a reduction in the heat transfer distance of PPT. These metal ions are known to possess excellent antimicrobial properties, which not only enhance the antimicrobial abilities of the composites but also support the photocatalytic activity of MXene. Additionally, the metals offer anti-inflammatory effects, promote blood vessel regeneration, and assist in the healing of wounds, making these materials more versatile in their functions.

### 5.4 Drug-doped MXene

MXene has attracted attention in building innovative drug delivery systems due to its surface area and outstanding photothermal conversion capability (Mohajer et al., 2022; Wan et al., 2022). Jin et al. (Jin et al., 2021) developed NIR and temperature-responsive MXene nanoribbon fibers (T-RMFs) containing vitamin E with controlled release capability. They prepared nanofibers consisting of MXene and polyvinylpyrrolidone-polyacrylonitrile (PAN-PVP) by electrostatic spinning technique, and then P copolymer coating was covered on the surface, and the prepared nanofibers had good wetting and diffusion effects. MXene endowed the nanofibers with excellent photothermal effects and antimicrobial activity. Activation of the thermo-responsive properties of MXene by NIR radiation resulted in the relaxation and release of vitamin E through the polymer-coated interface. Based on the results of the cellular evaluation, cells in the T-RMFs group revealed stronger cell attachment and proliferative capacity comparing with the control group, indicating their excellent wound-healing function. Zheng et al. (Zheng et al., 2022) synthesized the antibiotic ciprofloxacin (Cip) and Ti_3_C_2_T_x_ into a nano-complex and prepared an injectable temperature-sensitive hydrogel (Cip—Ti_3_C_2_TSG). *In vivo* and *in vitro* experiments confirmed that rapid and effective local chemical-photothermal bacterial ablation was achieved by accelerating Cip release under the NIR trigger. The physical membrane-disrupting effect of Ti_3_C_2_T_x_ nanosheets facilitated the penetration of Cip into the bacterial cells, and the sustained release of Cip prevented the rebound of bacteria after PTT. Xu et al. (Xu et al., 2021b) prepared a nanofiber membrane (MAP) with antimicrobial properties by electrostatic spinning using MXene, amoxicillin (AMX), and polyvinyl alcohol (PVA) as raw materials for the treatment of wound infections. MXene transfers NIR irradiation into thermal energy, leading to the upregulation of AMX release to overcome bacterial infection at the wound site. Under NIR irradiation, the antimicrobial rates against *E. coli* and *S. aureus* were 96.1% and 99.1%, respectively. *In vivo* experiments demonstrated that the MAP nanofiber membrane was efficient in improving the wound healing rate of infected mouse under laser irradiation. In another study, Sun et al. (Sun et al., 2021) utilized the photothermal conversion ability of MXene to increase the release of adenosine by NIR irradiation to maintain the activation signal around the injury site. When applied to a therapeutic animal model, it effectively promoted angiogenesis and facilitated wound healing. Zhang et al. (Zhang et al., 2022) designed a ROS- and pH-responsive composite system (MXene-TK-DOX@PDA nanoparticles) in which drugs were coupled to MXene-based nanocarriers via covalent bonding, which could avoid premature drug release, enhance loading capacity, and reduce adverse effects. In addition, coupling pH-responsive polydopamine on the surface of MXene-based nanoplatforms effectively improves the photothermal conversion ability. *In vitro* antimicrobial experimental data showed that the bactericidal rate against *E. coli* and *B. subtilis* reached 100% after 5 h.

In summary, MXene boasts a remarkable specific surface area and serves as an exceptional drug carrier. When combined with drugs, the antimicrobial properties of MXene can work in tandem to decrease the necessary dosage and enhance the therapeutic outcome. Furthermore, leveraging the photothermal properties of MXene allows for precise control over drug release rate and amount through the manipulation of light exposure. This approach mitigates the risk of drug overdose and promotes the wellbeing of patients.

## 6 Conclusion and outlook

Since the first ever finding of MXene by Yury Gogotsi’s group in 2011, MXene-based materials have drawn much attention in the energy and environment fields by the virtue of their tunable chemical compositions and unique physicochemical properties. In 2016, the application of MXene in the field of biomedicine attracted a great deal of attention, and since then, more and more research has been devoted to the exploration of MXene in biomedical field. MXene has been used in bioimaging, biosensing, and biomarker detection. Recently, MXene has induced extensive interest and research in antimicrobial applications. This article summarizes and discusses the research progress of MXene nanocomposites in antimicrobials, including the structure, synthesis method, biosafety, antimicrobial mechanism, and application in antimicrobial. The antimicrobial mechanisms of MXene are mainly (Turner et al., 2019): Physical damage to bacterial membranes due to the sharp edges of the nanomaterials (Pino et al., 2023); Promotion of ROS production and chemical damage caused by generating oxidative stress or charge transfer (Gao et al., 2021); Enhanced antimicrobial activity under photothermal and photodynamic synergies. In order to further enhance the antimicrobial capacity of MXene, scholars have tried to use the “addition method” to introduce metal nanoparticles, polymers, drugs, and other antimicrobial materials into MXene to synthesize MXene-based antimicrobial complex, hoping that multiple antimicrobial mechanisms will work together to reduce the emergence of bacterial drug resistance and fight against drug-resistant bacteria, and to promote the use of MXene in the antimicrobial field.

**FIGURE 5:**
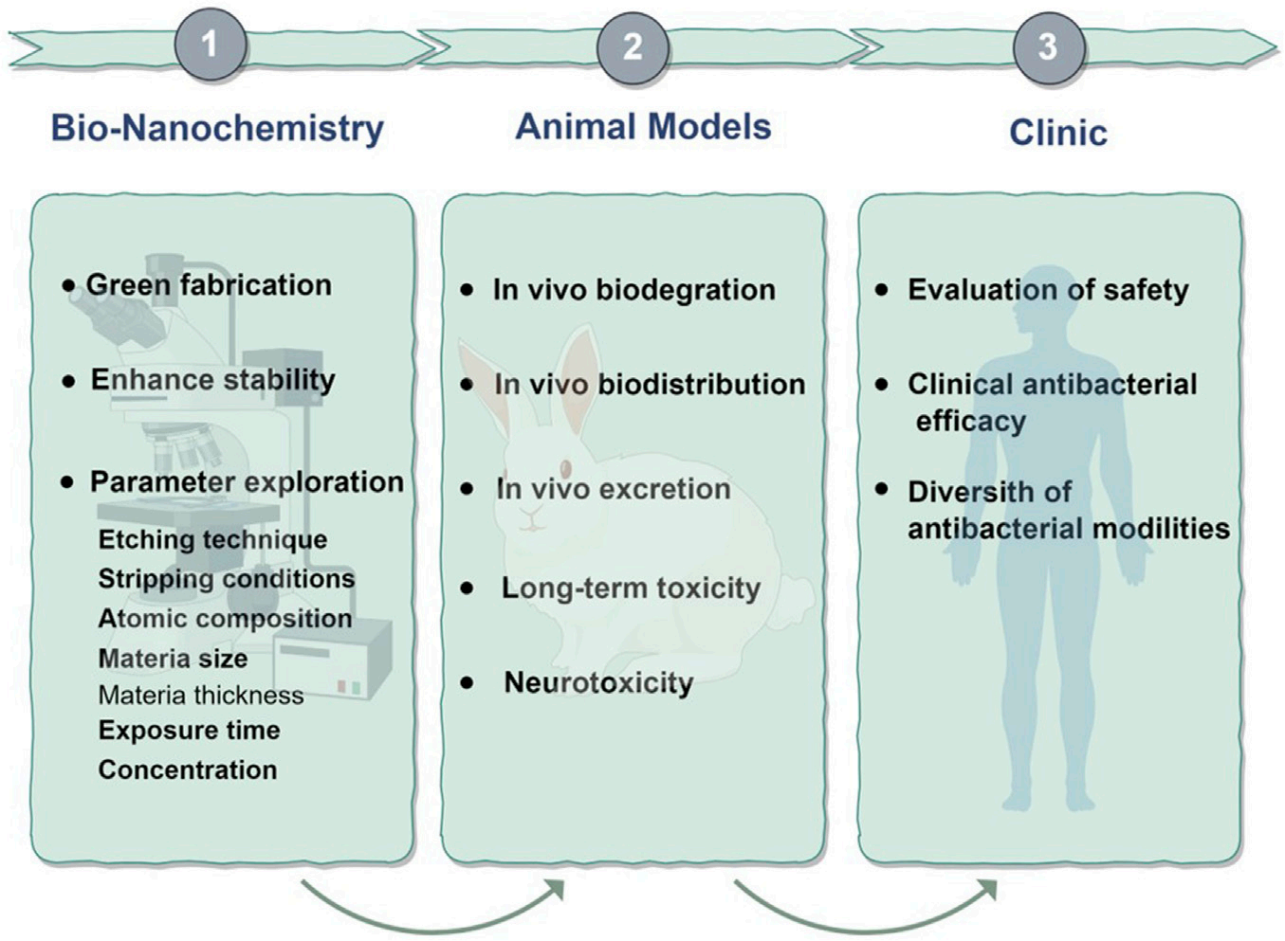
Perspectives for 2D MXene used in antibacterial.

Despite its many outstanding properties, the research of MXene in the biomedical field is still in the preliminary stage, and there are still many deficiencies and challenges compared to traditional 2D nanomaterials. First, MXene is unstable in humid environments or aqueous solutions and is prone to degradation or structural changes, which not only poses challenges to the preparation and storage of the material but also may affect its performance and safety in biomedical applications. How to synthesize MXene materials with high chemical stability is a key scientific problem that needs to be solved. In addition, the development of synthesis strategies based on green chemistry requires the study of more environmentally friendly preparation methods to minimize the harm to the environment. Secondly, MXene-based materials may accumulate in our body systems and long-term accumulation may cause potential toxicity. Although most of the studies have shown that MXene has a favorable biosafety profile, these studies are mostly short-term and have only been conducted in lower animal models such as zebrafish and mice, and studies on toxic effects in mammals are still very limited. In addition, the pharmacokinetic profile of MXene (including absorption, distribution, metabolism, excretion, and immune response) remains to be determined. Therefore, moving MXene-based materials from laboratory studies to commercialization for biomedical applications requires more comprehensive studies of their long-term effects *in vivo*, particularly on the immune system and reproductive system. Third, despite the progress made in applying MXene and its composite against pathogenic bacteria, many factors affect their antimicrobial effects (e.g., etching technique, stripping conditions, atomic composition, size, thickness, exposure time, concentration, etc.). More extensive research is needed to determine the appropriate parameters to aid in the diffusion and application of MXene before it can be realized for biomedical applications (Figure 5). Overall, MXene research in the biomedical field is still at an early phase, and there is a long way to run before it can be specifically applied in the clinical field.
